# The influence of menstrual risk factors on tumor characteristics and survival in postmenopausal breast cancer

**DOI:** 10.1186/bcr2212

**Published:** 2008-12-16

**Authors:** Chantal C Orgéas, Per Hall, Lena U Rosenberg, Kamila Czene

**Affiliations:** 1 Department of Medical Epidemiology and Biostatistics, Karolinska Institutet, Nobels väg 12A, Stockholm, SE-17177, Sweden; 2 Department of Clinical Sciences, Danderyd Hospital, Karolinska Institutet, Mörbygårdsvägen, Stockholm, SE-18288, Sweden

## Abstract

**Introduction:**

Hormonal factors are implicated in tumor progression and it is possible that factors influencing breast cancer induction could affect prognosis. Our study investigated the effects of menstrual risk factors on tumor characteristics and survival in postmenopausal breast cancer.

**Methods:**

We used a nationwide, population-based, case-case design of 2,640 Swedish women who were 50 to 74 years old and had postmenopausal breast cancer during 1993 to 1995. Follow-up was conducted until 31 December 2000. We used polytomous multiple logistic regression to investigate the relationships between menstrual factors (age at menarche, cycle length, irregular menstruation, lifetime number of menstrual cycles, and age at menopause), tumor characteristics (size, grade, estrogen receptor and progesterone receptor [PR] status, lymph node involvement, and histology), and Cox proportional hazards modeling for 5-year survival.

**Results:**

Younger ages at menarche were significantly associated with grade and lymph node involvement. Women with an age at menarche of 11 years or younger had a more than twofold excess risk of medium-grade (odds ratio [OR] = 2.05; 95% confidence interval [CI] 1.00 to 4.18) and high-grade (OR = 2.04; 95% CI 1.01 to 4.16) tumors. Early menarche significantly increased the risk of lymph node metastases. Survival was poorest in women with the earliest age at menarche, with a 72% increased risk of dying within 5 years after diagnosis (hazard ratio = 1.72; 95% CI 1.02 to 2.89). No significant associations were observed for other menstrual factors with tumor characteristics or survival.

**Conclusions:**

Age at menarche has a significant impact on breast cancer prognosis and survival. It remains to be established whether the associations are attributable to age at menarche directly or are associated with the early-life physiological events of breast development and carcinogenesis also taking place during childhood and puberty, as menarche is only the culmination of this series of events.

## Introduction

Haenszel [1] hypothesized that factors influencing breast cancer induction also affect prognosis. Estrogen promotes growth in breast cancer cell lines [2], and lower estrogen levels have been correlated with improved disease-free survival in postmenopausal breast cancer [3]. Tumor characteristics are important in determining survival [4], but they explain only a fraction of the variation observed in survival [5].

Several studies have generated support for Haenszel's hypothesis, with the confirmed association between obesity and poorer breast cancer prognosis [6-9]. To date, there have been conflicting results on whether menstrual risk factors for breast cancer influence tumor progression and survival in patients [9-19]. This may be partially due to variations in age categorizations for age-dependent risk factors, such as age at menarche and age at menopause, making interpretation difficult since there may be a critical time window of susceptibility during adolescent and puberty development that influences tumor initiation and progression.

The question of whether menstrual risk factors influence breast cancer prognosis is timely due to the decreasing age at menarche [20], with changes in nutrition [21] and lifestyle [22] patterns. Not many studies with detailed and varied information on tumor characteristics and survival outcomes have been conducted, nor have there been many large or population-based studies to address these research questions. Therefore, our study investigated the effects of menstrual risk factors on the prognostic significance of breast cancer in a population-based cohort of 2,640 patients with complete and detailed follow-up. This is the first study of its kind to address menstrual factors across a woman's lifespan, with detailed information regarding age at menarche, menstrual cycle length, irregular menstruation, lifetime menstrual cycles, and age at menopause and to equate to cumulative lifetime exposure to hormones acting on the breast.

## Materials and methods

### Study design

This study is an extension of a population-based case-control study among all women who were born in Sweden and who were 50 to 74 years of age between 1 October 1993 and 31 March 1995 and is described in detail elsewhere [23,24]. We used a case-case design in which we obtained odds ratios (ORs) and estimated hazard ratios (HRs), as measures of relative risk comparing breast cancer cases' categories of menstrual factors, to investigate the relationships between menstrual factors, tumor characteristics, and 5-year breast cancer survival.

### Participants

Women with incident primary invasive breast cancer were identified through the six Swedish Regional Cancer Registries and contacted by their doctors. Out of 3,979 women with a primary diagnosis of invasive breast cancer, 3,345 women (84%) participated in the study. The primary reason for nonparticipation was patient's or doctor's refusal due to patient ill health. Excluded patients had previous or other tumors (151 cases), noninvasive breast cancer according to patient records from the regional cancer registry (58 cases), a diagnosis outside the study period (19 cases), lack of patient consent (58 cases), premenopausal status (198 cases), being younger than 55 years with an unknown age at menopause (202 cases), missing age at first birth (5 cases), and missing height or recent weight (14 cases). The study included 2,640 eligible postmenopausal breast cancer patients of European descent.

### Protocol

The ethical review board at the Karolinska Institute (Stockholm, Sweden) and the six ethical review boards in other regions of Sweden approved the study. Prior to participation via a mailed questionnaire, written consent was obtained from all patients. The mean interval between diagnosis and data collection was 4.3 months (standard deviation of 1.5 months).

### Data collection and classification

With the exception of clinical data on tumor characteristics and follow-up data for survival outcomes, exposure and covariate data used in this study were derived from the case-control study questionnaire. In brief, data on sociodemographic, anthropometric, reproductive, and menstrual factors, use of oral contraceptives, and medical history (1 year prior to data collection) were collected by means of a postal questionnaire. Detailed information pertaining to the use of menopausal hormone therapy (MHT), including timing and type of hormones for each treatment episode, was requested along with a color chart that displayed all preparations ever marketed in Sweden and that was included with the questionnaire to facilitate recall. Additionally, approximately 50% of cases were contacted by telephone to complete missing or ambiguous responses, mainly on the use of MHT.

Menstrual factors assessed were age at menarche, age at menopause, irregular menstruation, cycle length, and lifetime number of menstrual cycles. Age at menarche was classified as not older than 11 years, older than 11 but not older than 13 years, older than 13 but not older than 14 years, and older than 14 years. Menopause was defined as the age at last menstrual period or age at bilateral oophorectomy if at least 1 year prior to data collection. To ensure an accurate classification of the true age at menopause, analyses for age at menopause were firstly restricted to women with known natural or surgical age at menopause who had not used MHT prior to menopause. Due to the large percentage of women with a missing true age at menopause (39%), we decided to use all women with an age at menopause and adjust our analyses for use and type of MHT. Age at menopause was grouped as younger than 50 years, 50 to 55 years, and older than 55 years. Irregular menstruation was either absent or present during the lifetime. Cycle length was classified as not more than 27.5 days per cycle, 28 days per cycle, or more than 28 days per cycle. Lifetime number of menstrual cycles was a created variable derived from all women with known values for age at menarche, age at menopause, parity, and cycle length and did not include those women with irregular menstruation or miscarriages and/or abortions. Lifetime number of menstrual cycles was classified as not more than 423 cycles per lifetime, more than 423 but not more than 500 cycles, and more than 500 cycles.

Information regarding tumor characteristics was retrieved from the medical records of all participants from surgical and oncological units throughout Sweden. Data pertaining to tumor characteristics included tumor size, grade (classified according to the Nottingham histological grade or Bloom-Richardson scale), estrogen receptor (ER) and progesterone receptor (PR) status, and lymph node involvement. Information on grade was not in routine use in Sweden during the study period and is therefore missing in 33% of patients.

The Swedish National Registration Number, a unique 10-digit number for each Swedish resident, was used to link the cohort with the Swedish National Population Register and the Swedish Cause of Death Register to obtain data on emigrations and on the dates and causes of death, respectively. The latter register covers all residents in Sweden and has been shown to accurately classify 98% of all breast cancer deaths [25].

### Statistical analyses

#### Tumor presentation

The significance of differences between tumor characteristics and menstrual factors was evaluated using frequencies with chi-square tests of association. All probability values of *P *less than 0.05 were considered significant. ORs with 95% confidence intervals (CIs) were calculated using polytomous multiple logistic regression [26], with tumor characteristics as the dependent variables, with the category of each tumor characteristics having the best prognosis as the reference group, and with the remaining categories as the outcome. Potential confounders were included in the models in a step-wise approach based on established biological knowledge of confounders particular to the associations of interest between menstrual risk factors and prognostic tumor characteristics, rather than solely based on a 10% percentage shift in the estimates.

#### Survival analysis

Follow-up time began on the date of breast cancer diagnosis and ended on the date of death, date of emigration, or date of study truncation or 5 years after the date of diagnosis, whichever occurred earlier. The outcome was breast cancer-specific deaths (International Classification of Diseases [ICD]-9: 174.9 and ICD-10: C50.9). One woman emigrated, 264 died from breast cancer, and 383 died from other causes during 12,290 person-years of follow-up.

Breast cancer mortality rates were calculated by menstrual factors as the number of breast cancer deaths per 100 person-years. Cumulative 5-year survival rates were calculated using the Kaplan-Meier method, and the significance of differences in survival was evaluated using the log-rank test. The Cox proportional hazards regression model was used to quantify the effects of menstrual factors on 5-year survival. The covariates chosen for multivariate analysis adjustments were based on biological associations deemed important in assessing menstrual factors and survival.

The menstrual prognostic factors in our study all occurred prior to the diagnosis of breast cancer. Therefore, any effects on tumor progression would have been mediated through the biological characteristics of the tumor itself. Hence, adjusting for any tumor characteristic variables in the Cox regression model would be incorrect as they did not confound the association between menstrual factors and breast cancer survival but rather acted as intermediates in the causal pathway. STATA^® ^version 9.2 (Statacorp, Texas, USA) was used for data analyses.

## Results

All menstrual factors were analyzed in relation to tumor characteristics and 5-year survival. Cycle length, total lifetime number of menstrual cycles, irregular menstruation, and age at menopause showed no significant trends, with most estimates close to unity. Therefore, only the results of survival analyses will be presented for these menstrual factors.

Background reproductive and menstrual factors of all cases in relation to age at menarche are summarized in Table 1. The majority of cases experienced menarche from more than 11 to not more than 13 years of age and from more than 13 to not more than 14 years of age (989 and 698 cases, respectively), with 9% missing age at menarche. In total, 264 deaths from breast cancer occurred. Fourteen percent of deaths occurred in women with the earliest age at menarche, 9% and 10% occurred in those with an intermediate age at menarche (from more than 11 to not more than 13 years and from more than 13 to not more than 14 years, respectively), and 8% in those with the oldest age at menarche.

**Table 1:** Distribution of background factors in postmenopausal women diagnosed with breast cancer in relation to age at menarche

	Age at menarche, years				*P *value^a^
		
	≤ 11	> 11 and ≤13	> 13 and ≤14	> 14	
	Column frequency, number (percentage)				
Total number of cases (n = 2,640)	167	989	698	542	
Background factors					
Menstrual cycle length, days					
≤27.5	35 (30)	183 (26)	125 (26)	93 (26)	
28	64 (55)	392 (55)	258 (54)	191 (54)	
> 28	17 (15)	136 (19)	96 (20)	71 (20)	0.877
Irregular menstruation					
No	159 (96)	39 (4)	18 (3)	518 (98)	
Yes	7 (4)	940 (96)	672 (97)	13 (2)	0.246
Age at first birth, years					
< 25	73 (51)	418 (50)	268 (45)	226 (49)	
≥25 and < 30	44 (31)	281 (34)	200 (34)	149 (32)	
≥30	27 (19)	136 (16)	129 (21)	86 (19)	0.240
Parity					
0	23 (14)	154 (16)	101 (14)	81 (15)	
1	30 (18)	215 (21)	154 (22)	105 (19)	
2	68 (41)	362 (37)	269 (39)	215 (40)	
3+	46 (28)	258 (26)	174 (25)	141 (26)	0.893
Pregnancy terminated < 6 months, nulliparous					
No	7 (88)	15 (38)	18 (49)	9 (45)	
Yes	1 (12)	25 (62)	19 (51)	11 (55)	0.079
Pregnancy terminated < 6 months, parous					
No	100 (71)	631 (76)	431 (73)	363 (79)	
Yes	40 (29)	197 (24)	160 (27)	95 (21)	0.069
Age at menopause, years^b^					
< 50	45 (31)	261 (31)	198 (34)	133 (28)	
50–55	86 (59)	520 (62)	344 (58)	299 (63)	
> 55	14 (10)	55 (7)	47 (8)	43 (9)	0.312
Ever use of MHT^c^					
No	105 (63)	646 (66)	483 (69)	370 (68)	
Yes	61 (37)	340 (34)	215 (31)	171 (32)	0.258
MHT use by general type					
Exclusive estrogen use	14 (9)	67 (7)	54 (8)	39 (8)	
Estrogen-progestin combined use	39 (25)	233 (25)	142 (21)	111 (21)	0.566
Diagnosed via mammographic screening					
Screening	102 (61)	546 (56)	399 (58)	301 (57)	
Other	64 (39)	436 (44)	288 (42)	228 (43)	0.484
Deceased at end of 5-year follow-up	27 (16)	134 (14)	101 (15)	74 (14)	0.802
Breast cancer deaths at end of 5-year follow-up (total = 264)	23 (14)	89 (9)	71 (10)	46 (8)	0.186

^a^Pearson chi-square tests of association between groups. ^b^Among women with a natural known menopause. ^c^Menopausal hormone therapy includes all treatments: exclusive estrogens, exclusive progestins, and combined estrogen-progestin therapy.

### Age at menarche and tumor characteristics

The associations of age at menarche and tumor characteristics of breast cancer patients are summarized in Table 2. Age at menarche was significantly associated with grade and lymph node involvement. ORs of the relation of age at menarche and tumor characteristics are summarized in Table 3. Only adjusted models are presented in the table as unadjusted estimates were virtually unchanged. Women with an age at menarche of not more than 11 years had a greater than twofold increased risk for tumors of medium grade (OR = 2.05; 95% CI 1.00 to 4.19) and high grade (OR = 2.04; 95% CI 1.01 to 4.16) compared with women with the oldest age at menarche with a low tumor grade. Similarly, women with intermediate ages at menarche had significantly increased risks for medium-grade tumors (OR = 1.47 [95% CI 1.00 to 2.15] in those older than 11 and not older than 13 years and OR = 1.74 [95% CI 1.15 to 2.62] in those older than 13 and not older than 14 years) and high-grade tumors (OR = 1.55 [95% CI 1.06 to 2.26] in those older than 11 and not older than 13 years and OR = 1.45 [95% CI 1.00 to 2.19] in those older than 13 and not older than 14 years). Women with earlier ages at menarche were also at a significantly increased risk for having tumors with lymph node involvement (OR = 1.49 [95% CI 1.02 to 2.19] and OR = 1.29 [95% CI 1.02 to 1.65] for the earliest age at menarche and for the age at menarche of more than 11 and not more than 13 years, respectively) compared with those oldest at menarche with no nodal involvement.

**Table 2:** Association of age at menarche with tumor-defined characteristics of breast cancer

	Age at menarche, years				
		
Tumor characteristic	≤11	> 11 and ≤13	> 13 and ≤14	> 14	*P *value^a^
Tumor size					
1–10 mm	47 (28)	258 (26)	183 (27)	153 (29)	
11–20 mm	76 (46)	414 (43)	302 (44)	250 (47)	
≥21 mm	43 (26)	304 (31)	199 (29)	129 (24)	0.188
Grade					
Low	12 (10)	98 (15)	66 (14)	71 (20)	
Medium	50 (44)	270 (40)	214 (46)	136 (39)	
High	52 (46)	304 (45)	188 (40)	141 (40)	0.037
ER status					
Positive	91 (73)	145 (20)	113 (23)	302 (79)	
Negative	34 (27)	564 (80)	381 (77)	78 (21)	0.309
PR status					
Positive	84 (69)	224 (32)	181 (37)	252 (68)	
Negative	38 (31)	473 (68)	305 (63)	121 (32)	0.250
Lymph node involvement					
Absent	104 (63)	630 (66)	456 (68)	374 (72)	
Present	60 (37)	329 (34)	217 (32)	146 (28)	0.052
Histology					
Ductal	124 (75)	692 (71)	503 (73)	383 (72)	
Lobular	24 (14)	118 (12)	78 (11)	62 (12)	
All other	18 (11)	165 (17)	108 (16)	87 (16)	0.509

^a^Pearson chi-square tests of association between groups. ER, estrogen receptor; PR, progesterone receptor.

**Table 3:** Relation of age at menarche to tumor-defined characteristics of breast cancer

	Age at menarche, years		
	Odds ratio (95% confidence interval)^a, b^		
Tumor characteristic	≤11	> 11 and ≤13	> 13 and ≤14
Tumor size			
1–10 mm			
11–20 mm	1.00 (0.65–1.53)	0.96 (0.74–1.25)	1.01 (0.76–1.33)
≥21 mm	1.05 (0.64–1.72)	1.43 (1.06–1.92)	1.32 (0.96–1.81)
Grade			
Low			
Medium	2.05 (1.00–4.19)	1.47 (1.00–2.15)	1.74 (1.15–2.62)
High	2.04 (1.01–4.16)	1.55 (1.06–2.26)	1.45 (1.00–2.19)
ER status			
Positive			
Negative	1.33 (0.82–2.16)	0.94 (0.68–1.29)	1.15 (0.83–1.61)
PR status			
Positive			
Negative	0.86 (0.54–1.36)	0.99 (0.75–1.30)	1.24 (0.93–1.66)
Lymph node involvement			
Absent			
Present	1.49 (1.02–2.19)	1.29 (1.02–1.65)	1.22 (0.95–1.58)
Histology			
Ductal			
Lobular	1.17 (0.68–2.01)	1.08 (0.76–1.52)	0.99 (0.68–1.43)
All other	0.68 (0.39–1.18)	1.09 (0.81–1.46)	0.92 (0.66–1.26)

^a^Reference group: age at menarche of more than 14 years, with the category of best prognosis within each tumor characteristic. ^b^Odds ratio estimates adjusted for body mass index at 18 years of age, age at first birth, age at diagnosis, and ever use and type of menopausal hormone therapy (never users, exclusive estrogen therapy, and combined estrogen-progestin therapy). ER, estrogen receptor; PR, progesterone receptor.

### Menstrual risk factors and survival

Kaplan-Meier survival curves showed significant differences in survival between the youngest and oldest ages at menarche (*P *value for log-rank test = 0.0466). As determined using a Cox model (Table 4), survival was poorest in women with the earliest age at menarche, with a 72% increased risk of dying within 5 years of diagnosis (HR = 1.72; 95% CI 1.02 to 2.89). Cycle length, total lifetime number of menstrual cycles, irregular menstruation, and age at menopause showed no significant trends in survival using the Kaplan-Meier method (data not shown) or Cox modeling (Table 4). Only adjusted estimates for survival are presented in Table 4 as unadjusted values were virtually identical.

**Table 4:** Breast cancer-specific five-year survival in relation to menstrual factors

Menstrual factors	Deaths	Mortality rate^a^	Hazard ratio (95% CI)^b^
Age at menarch, years^c^			
> 14	46	1.81	1.00 (reference)
> 13 and ≤14	71	2.19	1.26 (0.86–1.84)
> 11 and ≤13	89	1.93	1.14 (0.79–1.65)
≤11	23	2.99	1.72 (1.02–2.89)
Irregular menstruation			
No	253	2.16	1.00 (reference)
Yes	7	1.77	0.91 (0.43–1.93)
Cycle length, days			
≤27.5	39	1.79	1.00 (reference)
28	95	2.16	1.15 (0.78–1.68)
> 28	29	1.78	0.98 (0.60–1.61)
Total lifetime menstrual cycles, number			
≤423	58	2.10	1.00 (reference)
> 423 and ≤500	104	2.00	0.99 (0.71–1.38)
> 500	57	2.09	1.04 (0.72–1.52)
Age at menopause, years			
< 50	79	2.43	1.00 (reference)
50–55	138	2.18	0.92 (0.69–1.22)
> 55	17	2.22	1.04 (0.60–1.80)

^a^Breast cancer deaths per 100 person-years. ^b^All menstrual factor hazard ratio estimates adjusted for age at first birth, age at diagnosis, ever use and type of menopausal hormone therapy (never users, exclusive estrogen therapy, and combined estrogen-progestin therapy).^c^Age at menarche hazard ratio estimates additionally adjusted for body mass index at 18 years of age.

## Discussion

We found age at menarche to be significantly associated with tumor grade and lymph node involvement. Consistently, an age at menarche of 11 years or younger had the poorest survival. No associations were found with cycle length, irregular menstruation, lifetime number of menstrual cycles, and age at menopause with tumor characteristics or survival.

This is the first study to investigate menstrual factors of age at menarche, cycle length, irregular menstruation, lifetime number of ovulatory cycles, and age at menopause with tumor characteristics as the outcome. Our findings of significantly greater risks of higher-grade tumors and the presence of lymph node involvement with earlier ages at menarche appear to be novel. Only one study investigated hormone-related breast cancer risk factors and breast tumor proliferation, measured by the protein Ki-67 and mitotic count [16]. In contrast to our findings, those of that study found no significant association between tumor proliferation or mitotic count and age at menarche.

Age at menarche has previously been inconsistently associated with survival. Similar to our results are those of three other studies that also found an association between early age at menarche and reduced survival [10,12,19]. Caleffi and colleagues [10] studied women treated with modified radical mastectomy and found a significantly poorer survival with early age at menarche. In the study by Juret and colleagues [12], a proposed hypothesis suggested that the association may reflect a correlation with axillary nodal involvement, a hypothesis that our findings did not reject. The most recent study, by Trivers and colleagues [19], reported findings parallel to ours but their study was restricted to women less than 55 years of age and the association was evident only in premenopausal women when stratified by menopausal status.

Most other studies found no association with age at menarche and survival [9,11,15,17,18,27-30] or found an association opposite to our findings, with older age at menarche being associated with worse survival [13]. Possible explanations for the discrepancies in findings could be a difference in distribution of age at diagnosis, lack of adjustment for potential confounders, adjustment for tumor characteristics that are intermediates in the causal pathway of the association being addressed, and different categorizations of age at menarche.

It is well established that an early age at menarche is a risk factor for breast cancer [31]. The effects of age at menarche are linked to greater exposure to estrogens, which are promoters of breast cancer [32], as women with an early age at menarche have long-term increases in serum estradiol and lower serum sex hormone binding globulin (SHBG) concentrations than women with a late age at menarche [33]. These hormone levels prevail throughout the second and third decades of life [33]. We hypothesize that possible similar mechanisms act on breast carcinogenesis at earlier ages of menarche to influence the development and programming of tumors.

It should be emphasized that menarche is only the culmination of a series of hormonal, somatic, and anthropometric changes [34] and that early puberty, breast development, and menarche follow a naturally occurring process predetermined by a biological clock that, once initiated, turns on a rather independent process of breast development and maturation [35]. Puberty is a critical period in breast carcinogenesis [36] possibly explained by a very high number of terminal duct lobular units (that is, the functional units of the breast with the greatest proliferative activity [37] and the origin of most tumors [38]). Berkey and colleagues [39] hypothesized that rapid physical growth during adolescence gives less time for repair of DNA and thereby does permanent DNA damage with a carcinogenic potential. A recent study by Ahlgren and colleagues [40] showed that, after accounting for the growth patterns during childhood and adolescence, age at menarche was not related to risk of breast cancer. Similarly, we hypothesize that we cannot rule out the effects of puberty and growth during childhood and adolescence on the impressionable breast and that it is during this critical time window of susceptibility not only that breast carcinogenesis is initiated, but that tumor biology and prognosis are determined.

Furthermore, our findings that the total number of lifetime menstrual cycles a woman experiences is not associated with tumor characteristics and survival lend further support to the hypothesis of a critical time window of susceptibility acting on breast carcinogenesis and prognosis as opposed to the 'estrogen augmented by progesterone' hypothesis [41] of cumulative lifetime exposure to estrogens and progesterone through regular ovulatory cycles, since all estimates were close to unity.

Our results for age at menarche were adjusted for body mass index (BMI) at 18 years of age as BMI could be a confounder in the association of age at menarche and tumor characteristics and survival. Strictly speaking, BMI at 18 years is chronologically after menarche. However, in our study, we used this BMI as a proxy for childhood obesity. Thus, our results assume that no drastic changes in BMI occurred between childhood and 18 years of age. The timing of menarche is known to be affected by BMI as ovulation and menstruation require a threshold weight [22]. An increased body fat mass has been associated with early puberty and menarche [22,42]. Findings from a recent study substantiate the notion that body adiposity, possibly partly through hyperleptinemia, and insulin resistance are key contributors to observed variations in the timing of menarche [42]. Likewise, adiposity during childhood, at age 18, and premenopausally is associated with a decreased risk of premenopausal breast cancer, but the association with postmenopausal breast cancer remains inconclusive [40,43]. Ahlgren and colleagues [40] reported a significant reduction in risk of postmenopausal breast cancer with a larger BMI at age 14. Similarly, in another study, increased body silhouettes at ages 8 and around menarche were associated with a decreased risk of postmenopausal breast cancer [44]. However, Velie and colleagues [34] reported that few studies examined the effects of childhood growth in postmenopausal women exclusively, and that many studies do not yet contain older postmenopausal women. Additionally, these authors acknowledge the difficulties in extricating the effects of childhood body size from correlated adult body size with breast cancer risk. Similarly, as for risk, BMI is associated with prognosis [6-8]. Recent evidence suggests that excess body fat promotes the growth of breast tumors, with worse survival outcomes [6-8]. In our study, we cannot exclude the possibility that age at menarche could be an intermediary in the association between childhood BMI and prognosis. However, controlling for BMI at diagnosis did not attenuate the observed effects of age at menarche on tumor characteristics and survival. Our finding that age at menopause is not associated with survival is consistent with results from all previous studies [9,11,15,17,28,29], except one [13], which found that early or late age at menopause was associated with a poorer survival compared with women with menopause between the ages of 46 and 54 years.

### Strengths and limitations

Our study was population-based with a high response rate among cases (84%). Detailed information was available on menstrual risk factors in addition to other breast cancer risk factors. Sound information on tumor characteristics was collected, with only 2% and 3% of participants missing data on tumor size and lymph node involvement, respectively.

However, one limitation of our study was that nonparticipants had somewhat larger tumors on average and therefore these tumors were more likely to be receptor-negative with a higher proportion of lymph node involvement. We lacked information on tumor grade in 33% of cases. However, as all menstrual exposures assessed occurred well before the diagnosis of breast cancer, this would have had a negligible effect on our estimates. Similarly, receptor status was missing for 30% of cases, with estimates similar to those risks for developing ER-negative and PR-negative tumors. Receptor status was assessed at seven different laboratories, and several pathologists classified tumor size, lymph node involvement, and grade of differentiation. However, misclassification due to the nonstandardized analyses, together with the distribution of missing information, was certainly unlikely to be related to menstrual risk factors and is therefore nondifferential. For our analyses into lifetime number of menstrual cycles, we were not able to assess the effects of exclusivity of breastfeeding on breast cancer prognosis. In our study, we cannot exclude the possibility that our results are due to chance. However, we feel that this is unlikely since our findings were significant only for one menstrual factor (age at menarche) as we clearly showed significant findings, using two different methodologies and outcomes (which were biologically related), of poorer tumors and survival, and this is consistent with the interpretation of a poorer prognosis. All other menstrual factors addressed showed no significant finding for tumor characteristics or survival, with most estimated being around unity. Finally, we did not have childhood, pubertal, and adolescent somatic and anthropometric data to be able to disentangle whether the worse tumor characteristics observed in our study were due to these somatic and anthropometric factors themselves, or whether age at menarche, serves only as a proxy for these somatic and anthropometric factors: of which, these may be even more critical to breast cancer etiology.

## Conclusion

We found age at menarche to be significantly associated with grade and lymph node involvement and we found survival to be poorest in women with the earliest age at menarche. The finding of our study is timely due to the decreasing age at menarche in most developed countries and emphasizes the importance of potential early-life influences on breast tumor characteristics and survival.

## Abbreviations

BMI: body mass index; CI: confidence interval; ER: estrogen receptor; HR: hazard ratio; ICD: International Classification of Diseases; MHT: menopausal hormone therapy; OR: odds ratio; PR: progesterone receptor.

## Competing interests

The authors declare that they have no competing interests.

## Authors' contributions

CCO conducted all data management and statistical analyses, interpreted the data, and wrote the manuscript. PH revised the manuscript critically for important intellectual content and interpreted the data. LUR contributed to the acquisition of data and revised the manuscript critically for important intellectual content. KC conceptualized and designed the study, interpreted the data, and revised the manuscript critically for important intellectual content. All authors read and approved the final manuscript.
